# Vulnerabilities of protected lands in the face of climate and human footprint changes

**DOI:** 10.1038/s41467-021-21914-w

**Published:** 2021-03-12

**Authors:** Nawal Shrestha, Xiaoting Xu, Jiahui Meng, Zhiheng Wang

**Affiliations:** 1 State Key Laboratory of Grassland Agro-Ecosystem, Institute of Innovation Ecology, Lanzhou University, Lanzhou, China; 2 Institute of Ecology and Key Laboratory for Earth Surface Processes of the Ministry of Education, College of Urban and Environmental Sciences, Peking University, Beijing, China; 3 Society for Conservation Biology Nepal, Bagdol, Lalitpur, Nepal; 4 Key Laboratory of Bio-Resource and Eco-Environment of Ministry of Education, College of Life Sciences, Sichuan University, Chengdu, China

**Keywords:** Biodiversity, Conservation biology, Macroecology

## Abstract

Protected areas (PAs) play a pivotal role in maintaining viable populations of species and minimizing their habitat loss. Globally, there are currently over 200,000 PAs that cover approximately 15% of land area. The post-2020 global biodiversity framework aims to expand this coverage to 30% by 2030. However, focusing only on the percentage coverage of PAs without evaluating their effectiveness may fail to achieve conservation goals. Here, we use a multidimensional approach incorporating species, climate and anthropogenic vulnerabilities to assess the threat levels in over 2500 PAs in China. We identify nearly 10% of PAs as the most threatened PAs in China and about one-fifth PAs as hotspots of climate and anthropogenic vulnerabilities. We also find high climate instability in species vulnerability hotspots, suggesting an elevated likelihood of species’ extirpation therein. Our framework could be useful in assessing resiliency of global protected lands and also in selecting near optimal areas for their future expansion.

## Abstract (web summary)

Proected areas (PA) expansion is a major conservation goal, but its effectiveness is debated. Here, the authors propose a multi-dimensional framework to assess PA vulnerability and select areas suitable for expansion, demonstrating it for 2572 PAs in China under a low-emission scenario.

## Introduction

Human-induced global changes have increased the extinction risk of a large number of species on earth^1^. According to the report published by the Intergovernmental science-policy Platform on Biodiversity and Ecosystem Services (IPBES), the rate of species extinction in the past century has increased by 100 times compared to the average extinction over the past 10 million years^2^. The report further warns that if the present trend continues, up to 1 million terrestrial and marine species could be wiped out due to human activity. This reinforces the need for an immediate action to halt biodiversity loss globally.

Protected areas (PAs) are a globally accepted strategy for addressing this crisis^3–5^. They play a crucial role in minimizing habitat loss^6^ and maintaining sustainable population levels of species^7^. Although several challenges loom over their effective management, protected areas, nonetheless, serve as an effective tool for biodiversity conservation^7^. In response to rapid biodiversity loss globally, the number of protected areas has considerably increased in the last two decades. Since the United Nations Earth Summit in Brazil in 1992, the extent of protected areas has roughly doubled, with over 200,000 protected areas now covering approximately 15% of global land area^8^. The coverage of existing protected areas has, however, been found to be insufficient in addressing the current biodiversity crisis^9,10^ and the international community has been continuously working to increase the global coverage of protected areas.

The Strategic Plan for Biodiversity 2011–2020 adopted by the Conference of the Parties (COP) to the Convention on Biological Diversity (CBD) includes 20 targets (also known as the Aichi Biodiversity Targets), among which, Target 11 states that, “By 2020, at least 17% of terrestrial and inland water areas, and 10% of coastal and marine areas, especially areas of particular importance for biodiversity and ecosystem services, are conserved through effectively and equitably managed, ecologically representative and well connected systems of protected areas and other effective area-based conservation measures, and integrated into the wider landscapes and seascapes”^11^. The draft report of the post-2020 global biodiversity framework, which will be taken up at a United Nations biodiversity summit in China, further suggests expanding protected areas to at least 30% of land areas by 2030^12^. However, one of the greatest challenges for expanding protected areas is to select the right areas for conservation. Globally there are many instances where areas that do not have the highest conservation need are designated as protected areas to fulfill the Aichi target^13^. Recently, conservation biologists have argued that selecting the right areas for conservation is more important than the percentage coverage of protected areas^14^, which means that merely fulfilling the Aichi target (17% coverage of PAs) or the post-2020 biodiversity target (30% coverage of PAs) without evaluating the conservation impact of the designated protected areas is less meaningful to achieving reliable biodiversity conservation^13^. Studies have shown that selecting areas by focusing only on quantity are largely ineffective for biodiversity conservation^15,16^. Therefore, instead of focusing only on fulfilling the quantitative coverage target, countries should preemptively evaluate the efficiency of the designated protected areas to see if the areas that are under protection also have the highest conservation need^13^.

Climate change and anthropogenic pressure are two global challenges that pose immense threat to biodiversity^17^. Climate change is projected to greatly increase species vulnerability, with more species experiencing contraction in suitable habitats than those experiencing habitat expansion in the future^2^. Similarly, anthropogenic pressure, which is projected to increase further in the future, is likely to displace species from their natural habitat causing serious declines in their populations^18^. In this scenario, the survival or extinction of a species will entirely depend upon how effectively these two global threats are addressed in the spatial planning and management of future protected areas. Although protected areas provide buffer against direct human intervention, climate change will inevitably affect species’ range size^19^. Areas that are suitable for species now may either be climatically unsuitable or may be under increased human pressure in the future. Therefore, the vulnerability of a protected area needs to be evaluated by considering these two dimensions. Assessing the tempo of change in climate and anthropogenic pressure could help us to gauge actual threat within protected areas. A protected area experiencing high climate change and high anthropogenic pressure is more vulnerable compared to a protected area experiencing mild climate change and low anthropogenic pressure. Principally, the former protected area needs more stringent protection measures than the latter because species inhabiting the former are more likely to be threatened in the future due to high intensity of climate change and anthropogenic pressure therein. In an era when climate change and anthropogenic pressure are posing great threat to species’ survival, it is, therefore, imperative to take into consideration biodiversity value (i.e., number of threatened species) within protected lands together with the dynamics of climate change and anthropogenic pressure to identify vulnerable areas that require immediate conservation attention.

Here, we develop a framework to quantify the level of threat in over 2500 protected areas in China incorporating three dimensions: species vulnerability, climate vulnerability and anthropogenic vulnerability. We classify these protected areas into different threat categories according to their vulnerability scores and identify areas that require the highest conservation attention. Our assessment identifies 17% of PAs as the hotspots of climate and anthropogenic vulnerabilities, and 23% of PAs as the hotspots of species vulnerabilities. These PAs require the highest conservation attention. Interestingly, the species vulnerability hotspots (SVH) have experienced higher climate change since the 1960s compared to species vulnerability coldspots (SVC), which indicates that the SVH have remained climatically unstable since the past. This is very concerning from a conservation perspective, as climate is likely to change at the previous pace. Our findings suggest that habitat loss due to increased anthropogenic pressure could likely be a major threat to species in most protected areas in China. We recommend that future assessments should incorporate climate change and anthropogenic threats together with species vulnerability to maximize the conservation efficiency of protected areas. Our framework is useful for evaluating the vulnerability of global protected lands and also for selecting resilient areas for expanding PA coverage to meet the post-2020 global biodiversity target^12^.

## Results

### Spatial patterns of climate, anthropogenic, and species vulnerabilities

**Fig. 1:**
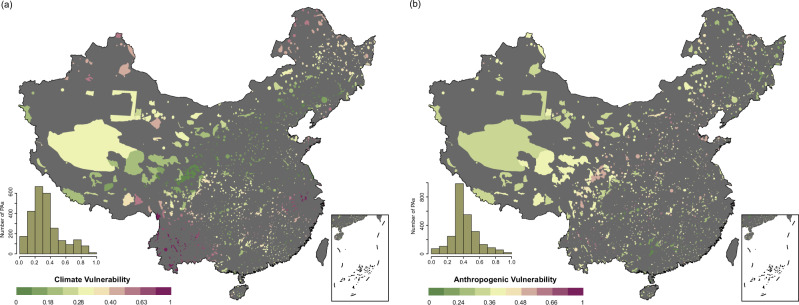
Spatial patterns of climate and anthropogenic vulnerabilities across protected areas (PAs) in China. **a** climate vulnerability and **b** anthropogenic vulnerability. Values close to 1 represented by warmer color indicate high climate or anthropogenic vulnerability. The insets in each subfigure show the frequency distribution of respective vulnerability scores.

The intensity of climate change is relatively high in the protected areas in southwestern, eastern, northern, and western China (Fig. 1a). These regions include Yunnan, Sichuan and parts of the Qinghai-Tibetan Plateau in southwestern China, Anhui and Jiangxi in eastern China, Heilongjiang and Inner Mongolia in northern China and Xinjiang in western China. The protected areas in western and northern China have experienced the highest climate warming in the last 60 years (Supplementary Fig. 1). On the contrary, the protected areas in southeastern and northeastern China have experienced low to mild warming. About one-third protected areas have experienced average warming higher than 1.5 °C in the last 60 years (Supplementary Fig. 1).

The intensity of human footprint change is the highest in the protected areas in northern, southwestern and eastern China (Fig. 1b). These regions include Heilongjiang, Liaoning and Hebei in northern China, Yunnan and Sichuan in southwestern China and Jiangxi and Anhui in eastern China. PAs in western China have relatively lower intensity of human footprint changes than other regions. About one-fourth protected areas have experienced an increase of human footprint >4 during the studied period (Supplementary Fig. 1).

**Fig. 2:**
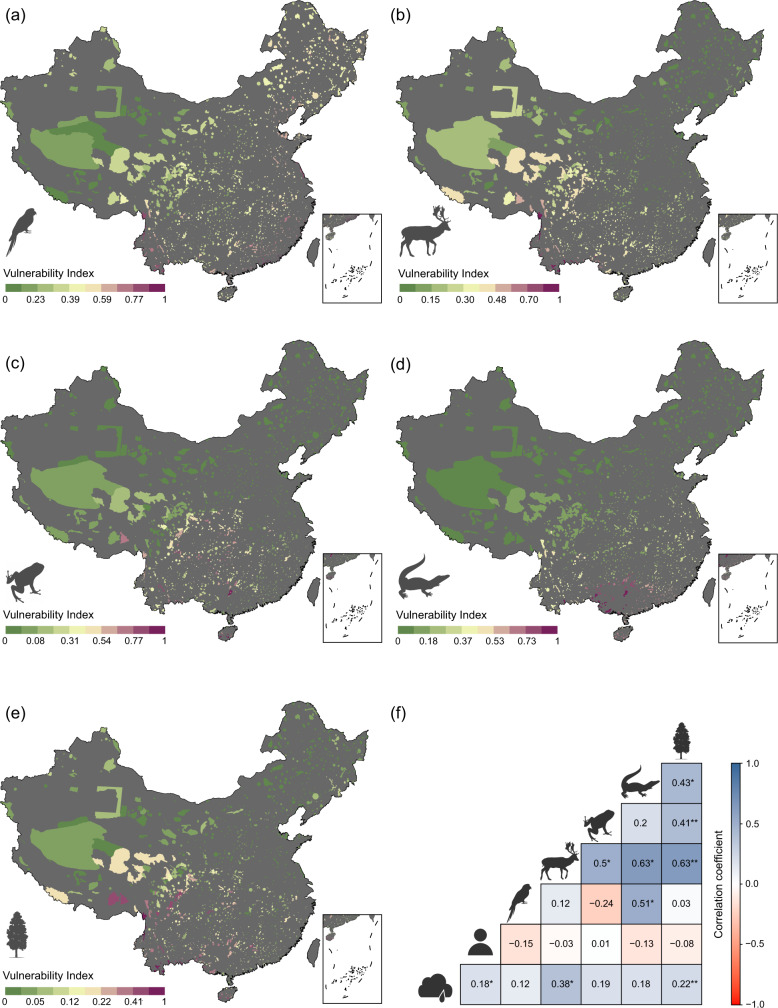
Spatial patterns of species vulnerabilities across protected areas in China. **a** birds, **b** mammals, **c** amphibians, **d** reptiles and **e** plants. Vulnerability index close to 1 represented by warmer color indicates high species vulnerability (i.e., high diversity of threatened species in general, or a high number of highly threatened species, such as critically endangered, or both). **f** Pearson’s correlation coefficients between climate, anthropogenic and species vulnerabilities. The significance of the correlation tests was computed using modified *t*-test. **p < 0.01, *p < 0.05. Values without an asterisk (*) are not significant.

The vulnerable species of plants and vertebrates mainly occur in the protected areas in southern and southwestern China (Fig. 2). The concentration of vulnerable birds, however, expands towards northern and eastern China as well (Fig. 2a). Yunnan, Sichuan, Guizhou and Guangxi provinces and the southeastern fringe of the Qinghai-Tibetan Plateau harbor the greatest number of vulnerable species of all groups. Northern and western China have relatively fewer number of vulnerable species compared to southern China.

The vulnerability score of one species group do not fully correspond to those of the other groups (Fig. 2f). For example, the Pearson’s correlation coefficient (*r*) between the vulnerability scores of birds and plants is only 0.03 and is non-significant (*p* = 0.807). The highest correlation is observed between the vulnerability scores of mammals and plants (*r* = 0.63, *p* < 0.001) and mammals and reptiles (*r* = 0.63, *p* = 0.045). Similarly, the vulnerability scores of all vertebrate groups and plants are weakly correlated with both climate and anthropogenic vulnerabilities. However, unlike climate vulnerability, anthropogenic vulnerability is not significantly correlated with species vulnerability of any groups (Fig. 2f). The correlation coefficient between climate and anthropogenic vulnerabilities is also very weak (*r* = 0.18, *p* = 0.021).

### Vulnerability hotspots

The climate vulnerability hotspots (i.e., PA with significantly high climate vulnerability: one-tailed test, *p* < 0.05) occur in southwestern and eastern China, particularly in Yunnan, Anhui, Zhejiang and northern Jiangxi (Supplementary Fig. 2a). Anthropogenic vulnerability hotspots (i.e., PA with significantly high anthropogenic vulnerability: one-tailed test, *p* < 0.05) spread all over China except western and central China (Supplementary Fig. 2a). The protected areas in Yunnan are the mutual hotspots of climate and anthropogenic vulnerabilities. The climate and anthropogenic vulnerability hotspots together account for about 17% protected areas in China.

The species vulnerability hotspots (i.e., PA with significantly high species vulnerability: one-tailed test, *p* < 0.05) are mostly concentrated in southern China (Supplementary Fig. 2b). However, the vulnerability hotspots of different groups are spatially dispersed. The vulnerability hotspots of mammals, for example, mainly occur in southwestern (Tibet, Yunnan, Sichuan) and southern (Guangxi) China, while the vulnerability hotspots of birds mainly occur in southern (Yunnan, Guangdong) and eastern (Fujian) China. Compared to other groups, the vulnerability hotspots of reptiles occupy very narrow geographical extent and occur mostly in southern China (Guangxi). The species vulnerability hotspots of vertebrates and plants together account for about 23% protected areas in China.

**Fig. 3:**
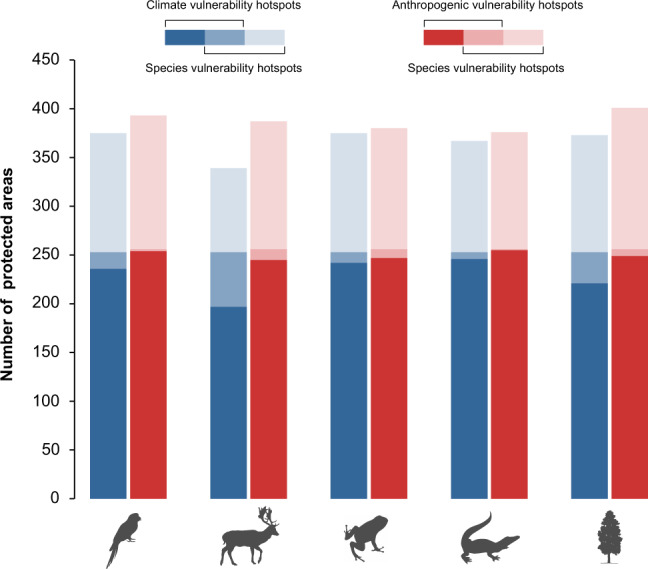
Overlap of species vulnerability hotspots of five species groups with climate and anthropogenic vulnerability hotspots. The hotspots were identified by randomizing respective vulnerability scores of each protected area (PA) 1000 times and keeping a track of where the observed value was greater than the randomized value. If the observed value was within the highest 5% of the null distribution (one-tailed test) for that PA (i.e., at least 950 times higher than the 1000 randomized value), it was identified as hotspots.

The species vulnerability hotspots of each group do not completely overlap with one another (Supplementary Fig. 2c). The overlap between the vulnerability hotspots of birds and amphibians, for example, is only 1% (3 protected areas). The highest overlap is between the vulnerability hotspots of mammals and plants (28%). Similarly, the vulnerability hotspots of all vertebrates and plants only partly overlap (0–17%) with the climate and anthropogenic vulnerability hotspots (Fig. 3 and Supplementary Fig. 2c). The overlap is less than 10% for most groups except that between mammals and climate hotspots, which is 17% (Fig. 3 and Supplementary Fig. 2c). About 7–56 protected areas are the mutual hotspots of species vulnerability and climate vulnerability (Fig. 3 and Supplementary Fig. 3), while 1–11 protected areas are the mutual hotspots of species vulnerability and anthropogenic vulnerability (Fig. 3 and Supplementary Fig. 4).

### Classification of protected areas

**Fig. 4:**
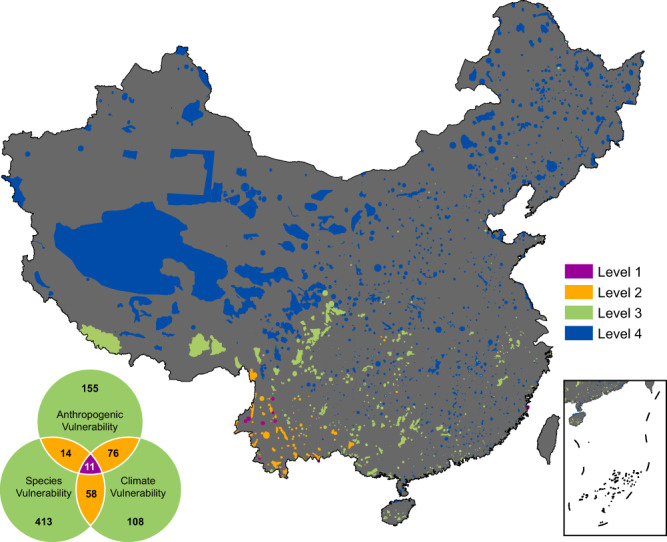
Classification of Chinese protected areas (PAs) based on three vulnerability indices: climate vulnerability, anthropogenic vulnerability, and species vulnerability. The PAs in magenta represents the hotspots identified by all three indices (Level 1), the PAs in orange represents the hotspots identified by two indices (Level 2) and the PAs in green represents the hotspots identified by only one index (Level 3). Protected areas that lie outside the hotspots of these three indices are shown in blue (Level 4). Numbers in the Venn diagram are the total number of protected areas in each category.

We categorized all protected areas into four levels ranging from highly vulnerable to least vulnerable based on the number of overlaps of the three vulnerability indices, i.e., climate, anthropogenic and species vulnerabilities (see Methods). We find that 11 protected areas mostly occurring in southwestern China (Yunnan) belong to the “Level 1” category, while 148 protected areas belong to the “Level 2” category (Fig. 4). The “Level 2” protected areas are mainly concentrated in southwestern China (Yunnan, Chongqing), central China (Hunan) and parts of southern China (Guangxi). The “Level 1” and “Level 2” protected areas together account for about 7% of all protected areas and are identified as the most vulnerable protected areas in China. These protected areas are climatically unstable, have high anthropogenic pressure and high species vulnerability, and therefore, require the most stringent protection measures. The “Level 3” protected areas occur mostly in southern, southwestern and eastern China, while the “Level 4” protected areas occur throughout China with high concentration in northern and western China. The “Level 4” protected areas have the least number of vulnerable species and also have low anthropogenic and climate vulnerabilities.

### Climate and human footprint changes in species vulnerability hotspots and coldspots

**Fig. 5:**
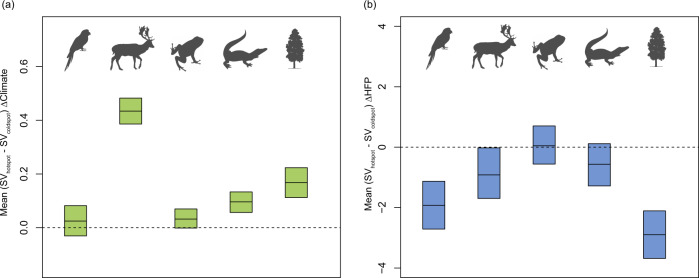
Difference in climate change (ΔClimate) and human footprint change (ΔHFP) between species vulnerability (SV) hotspots and coldspots of respective groups. **a** difference in climate change between species vulnerability hotspots and coldspots and **b** difference in human footprint change between species vulnerability hotspots and coldspots. Central solid lines indicate mean differences between a random hotspot point and a random coldspot point and the upper and lower bounds represent 90% confidence interval calculated with 1000 bootstrap replicates.

The intensity of climate change (ΔClimate) is significantly higher (*p* < 0.05) in species vulnerability hotspots (SVH) than in species vulnerability coldspots (SVC) of all the studied groups (Fig. 5). On the contrary, the intensity of human footprint change (ΔHFP) is significantly lower (*p* < 0.05) in SVH than in SVC (Fig. 5).

## Discussion

Climate is expected to warm rapidly in the future^20^ and this will inevitably affect species’ future distributions^2,19^. Based on our assessment, Chinese protected areas have already experienced climate warming of 0.7–3.7 °C in the past 60 years with two-third protected areas experiencing average warming higher than 1 °C and one-third experiencing average warming higher than the Paris Agreement temperature limit of 1.5 °C^21^. Similarly, under the most optimistic emission scenario (i.e., RCP2.6), nearly one-third Chinese protected areas are expected to experience further warming higher than 1.5 °C in the future (Supplementary Fig. 1). These numbers will likely increase in the other emission scenarios. The IPCC special report on climate change suggests that at 1.5 °C warming, the climatically determined geographic range of 6% of insects, 8% of plants and 4% of vertebrates will be reduced by more than half^22^. Other studies have shown a direct association of climate warming with the local extinction of bumble bees in North America and Europe^23,24^ and declines in populations of terrestrial birds and mammals globally^25^. Future climate warming is likely to cause population and distribution range declines of species in Chinese protected areas too and such declines are likely to be higher in regions where the temperature will surpass species’ observed tolerances^24^.

Interestingly, the magnitude of climate change is significantly higher (*p* < 0.05) in species vulnerability hotspots (SVH) than in species vulnerability coldspots (SVC). Although the observed pattern does not suggest a mechanistic link between high climate change and SVH, it does indicate that the SVH have remained climatically unstable in the last 60 years, which is more concerning from a conservation perspective because SVH currently harbors higher number of threatened (CR, EN, VU) species. If we assume this warming trend to increase linearly as it did in the past (and it is highly likely it might), the range sizes of species would shrink further, making species in SVH even more threatened. In other words, the impacts of future climate warming on threatened species are likely to be more severe in species-rich protected areas than in species-poor protected areas, which may lead to disproportionately high risks of species extirpation in species-rich protected areas.

Increase in human pressure have been shown to directly influence the local population of species. For example, Wan et al.^17^ have provided quantitative evidence showing local extinction of mammals in China associated with intensified human disturbance and extreme temperature change. Previous analyses have also shown that in regions with human footprint values higher than 4, species are far more likely to be threatened due to habitat loss^26,27^. Based on our assessment, about 90% PAs in China exceed this threshold currently and the human footprint increase since the past is more than 4 in about one-fourth PAs (Supplementary Fig. 1). This indicates that habitat loss due to increased anthropogenic pressure could likely be a major threat to species in most protected areas in China. Particularly, PAs in the southern, eastern and northern China are more susceptible to this threat as these regions have experienced increased anthropogenic pressure since the past.

Unlike climate change, the intensity of human footprint change is, however, significantly lower (*p* < 0.05) in SVH than in SVC. Nonetheless, based on the current scenario, human pressure may still be a strong limiting factor on species diversity, particularly in SVH because SVH mostly occurs in regions with mild climates (see Supplementary Fig. 5), which are also preferred by humans. Disentangling the relative roles of climate and anthropogenic pressure on species diversity is, however, beyond the scope of the present study and further studies are needed to evaluate their independent contributions.

Incongruence between the diversity hotspots of different taxa has been frequently documented^28,29^. Consistent with previous studies, we also find very low coincidence between the vulnerability hotspots of different species groups, which indicates that the priority area for one group may not be fully representative for other groups^28^. For example, a protected area designed to protect mammals may only be partially effective in protecting birds or reptiles and vice versa. Most importantly, we find that both climate vulnerability hotspots and anthropogenic vulnerability hotspots show very negligible overlap with the species vulnerability hotspots of all taxa (Supplementary Figs. 3 and 4) and this indicates that focusing only on species vulnerability hotspots for spatial prioritization, which is a common global practice, may increase the extinction risks of some rare species surviving in species vulnerability coldspots^30^ that are climatically and anthropogenically more vulnerable.

Although PAs are established to conserve biodiversity and ecosystems, these targets are not fully taken into consideration while delineating PA boundaries. Due to difficulties in land acquisition, marginal lands that have the least pressure of conversion are often selected for PA expansion^31^. This results in establishing protected areas in regions that are either unimportant to biodiversity or have the least conservation need^13,32^. However, since the past decade, there has been a shift from this ad hoc practice to a more robust systematic conservation planning^33,34^. With regard to the multifaceted responses of biodiversity to various environmental factors, conservationists have realized that prioritizing areas using biodiversity alone may not be sufficient^30^. This has encouraged the surge of studies incorporating land tenure^3^, ecosystem services^35^, human population density^36^ and climate connectivity^37^ in systematic conservation planning. Our framework adds another dimension to this global conservation strategy and emphasizes the necessity of considering climate and anthropogenic vulnerabilities together with species vulnerability for prioritizing areas for conservation.

The current framework, which incorporates species vulnerability, climate vulnerability and anthropogenic vulnerability for assessing the vulnerability of PAs, has two-fold benefits. First, it helps to identify vulnerable areas that are not only the hotspots of threatened species but also the hotspots of climate change and anthropogenic pressure. This information is useful to allocate conservation priorities. For example, our assessment on PAs in China revealed that about 7% PAs (Level 1 and Level 2), located in southwestern (Yunnan, Chongqing), central (Hunan) and parts of southern China (Guangxi) are highly vulnerable (see Fig. 4). The species in these PAs are likely at risk due to high climate change and habitat loss, and stringent protection measures are urgently required to maintain their effectiveness. Equally important are the climate and anthropogenic vulnerability hotspots located in eastern and northern China. Second, the framework can assist in identifying near optimal areas (those with high biodiversity, fairly stable climate and low anthropogenic pressure) for future expansion of protected areas.

Although our climate vulnerability metric principally corresponds to Carroll et al.’s climate connectivity measure^37^, our study differs from previous studies in two ways. First, we estimated the climate vulnerability of each PA by measuring the intensity of past climate change, which is based on actual observed data. We believe that our estimates of climate vulnerability offer realistic measure of climate threat in respective PAs. Second, we simultaneously assessed the threats in PAs using multiple dimensions, including biodiversity value, climate change and anthropogenic pressure. This likely provides a more balanced approach to estimating vulnerability of respective PAs compared to those based on a single dimension. However, we acknowledge that our framework should not be viewed as a replacement to previous frameworks but complementary to them. Planners should take them as sources of complementary information and use them subjectively in conservation planning.

Our findings provide useful insight for delineating PA boundaries in future conservation planning in China. China’s ambitious Ecological Red Line (ERL) conservation initiative^38^ was initiated in 2011 to protect all its rare and endangered species and their habitats, and it involves assessment from multiple dimensions, including biodiversity, ecosystem services, resiliency to natural disasters, and soil erosion^39^. We recommend that the future expansion of PAs under the ERL conservation initiative should consider climate change threats as well as anthropogenic pressure to appropriately gauge the vulnerability of prioritized areas. We also recommend establishing PAs in regions that are climatically less vulnerable as such areas offer increased opportunities for species’ survival and persistence^37^. Our framework is useful for identifying vulnerable PAs globally so that timely proactive measures could be taken to keep the biodiversity and ecosystem intact therein. It could be equally useful in assessing resiliency of global protected lands and in prioritizing areas for their systematic expansion, to meet the post-2020 global biodiversity target^12^.

## Methods

### Spatial map of Chinese protected areas

The database of protected areas (PA) distribution in China and a digitized spatial map thereof were compiled from Zhao et al.^40^ and Zhang et al.^41^. In total, we obtained the information of 2622 protected areas in China, which also included marine reserves. In order to evaluate the representation of terrestrial protected areas, we excluded marine reserves from our analyses. We also excluded Taiwan because we did not have the spatial distribution data for nature reserves in Taiwan. Finally, we had the boundary information of 2572 protected areas covering about 15.2% land area in China.

### Species’ range maps

Range maps of threatened vertebrates (birds, mammals, amphibians and reptiles) were obtained from the IUCN’s Red List^42^. Distribution data of threatened plants were compiled from Flora of China, Atlas of woody plants in China, provincial and local floras, checklists of nature reserves, various inventory reports across China and peer-reviewed papers. We obtained the information for critically endangered (CR), endangered (EN) and vulnerable (VU) species. The conservation status of vertebrates was obtained from IUCN Red List^42^, while that of plants was obtained from Qin et al.^43^. In total, we obtained the distribution information of 103 birds, 86 mammals, 134 amphibians, 50 reptiles, and 2983 plants in China (see Supplementary Data 1). We estimated the number of species in each PA by overlaying the map of PA with the species’ range maps in ArcGIS 10.2 (ESRI, Redlands, CA). In order to validate the distribution of species, we further verified the presence of species in respective PA by checking their inventory reports.

### Human footprint data

In order to measure the extent of human pressure on the protected areas, we obtained the most comprehensive global map of human pressure i.e., human footprint (HFP) from https://wcshumanfootprint.org. The human footprint measures the cumulative impact of direct pressures on environment from human activities and is based on data from built environments, agricultural lands, pasture lands, human population density, night-time lights, railways, roads and navigable waterways^44^. It is one of the most complete and finest terrestrial datasets on cumulative human pressure on the environment. The human footprint maps of two time periods (1993 and 2009) are available at present. We downloaded the maps of both time periods at the spatial resolution of 1 km × 1 km to quantify the change in human pressure within Chinese protected areas over a 16-year period. It should, however, be noted that any point estimate of the change in HFP might include errors due to the resolution and reliability of the component layers. For example, one of the components of HFP is the night-time lights, which changed over time from incandescent to mercury vapor to light emitting diode. This means that the change in night light is due to more than development. As a result, the systemic bias in regional economy could likely cause low HFP in wealthy as compared to rural areas. While this issue does not invalidate our analyses, such comparisons should be applied with caution.

### Climate data

In order to represent the climatic conditions of the past and the present, we obtained 20 years climate data comprising monthly minimum temperature, monthly maximum temperature and monthly precipitation for two time periods (past: 1961–1970 and present: 2010–2019). We used 10 years window for each time period to capture the variability in climatic conditions in order to prevent over- or underestimation of the past and present climate. In total, we obtained 12 months × 10 years × 3 variables × 2 time periods = 720 global raster layers from the Climate Research Unit (CRU TS v. 4.04) database (http://www.cru.uea.ac.uk/data) at the spatial resolution of 0.5° × 0.5°^45^. We then calculated the monthly mean values for the three variables for each time period separately. From these monthly mean values, we estimated mean annual temperature (MAT), mean temperature of warmest quarter (MTWQ), mean temperature of coldest quarter (MTCQ), mean annual precipitation (MAP), precipitation of driest quarter (PDQ) and precipitation of wettest quarter (PWQ) for each time period using biovars function in the R package ‘dismo’^46^. We then estimated the average value of climate variables in each PA using zonal.stats function in the R package ‘spatialEco’^47^. In order to reduce dimensionality and collinearity of the 6 climate variables, we performed principal component analysis (PCA) using prcomp function in R v4.0.2^48^. Following Carroll et al.^37^, we used climate data for both past and present based on the first 2 PCA axes, which explained 89.4–89.8 % of the variance (Supplementary Tables 1-2). Additionally, we also estimated the change in mean annual temperature to identify PAs that have experienced climate warming higher than the Paris Agreement threshold of 1.5 °C. All the analyses were performed in R version 4.0.2^48^.

### Vulnerability mapping

We calculated three indices of vulnerability within each protected area: (i) species vulnerability, (ii) anthropogenic vulnerability, and (iii) climate vulnerability. In order to measure species vulnerability, we first assigned numerical value to each IUCN threat category using a geometric progression^49,50^. We gave scores of 2, 4, and 8 to species belonging to categories VU, EN, and CR, respectively. We, then, summed the score of all species in each PA and standardized the value to the range of 0–1 using minimum–maximum normalization. We performed these steps separately for birds, mammals, amphibians, reptiles and plants to calculate the vulnerability score of each group. We also calculated the cumulative score by combining the total scores of all five groups. Values close to 0 indicated low species vulnerability and values close to 1 indicated high species vulnerability. Although species diversity is highly correlated with the species vulnerability metric used herein (Pearson’s correlation coefficient = 0.94 − 0.99, *p* < 0.001), the species vulnerability metric is more useful from a conservation perspective because it offers trade-off between proactive (i.e., protecting high biodiversity) and reactive (i.e., protecting threatened species) conservation practices. While proactive approaches are useful to keep the ‘common species common’, reactive approaches are necessary to prevent the extinction of already threatened species^51^. Our species vulnerability metric captures both species diversity and the threat level of a species. Therefore, high species vulnerability may either mean high diversity of threatened species (CR, EN, VU) or higher number of highly threatened species, such as “Critically Endangered” or both.

In order to measure anthropogenic vulnerability, we first estimated the average HFP value in each PA using the zonal.stats function in the R package ‘spatialEco’^47^. We did this separately for 1993 and 2009. We, then, subtracted the average HFP value in 1993 with the average HFP value in 2009 to estimate the change in HFP (ΔHFP) in each PA. Finally, we standardized the ΔHFP value to the range of 0–1 using minimum–maximum normalization. Value close to 1 indicate high anthropogenic vulnerability (i.e., greater increase in human pressure since the past), while value close to 0 indicate low anthropogenic vulnerability.

In order to measure climate vulnerability, we estimated the change in climate in each PA by calculating the Euclidean distance between the past and present distributions in a 2-dimensional climate space (i.e., PCA1 and PCA2 axes). Larger distance between the past and present distributions means higher deviation from original climate and hence represent high climate vulnerability therein. We standardized the climate change value for each PA to the range of 0–1 using minimum–maximum normalization. Value close to 1 indicate high climate vulnerability (i.e., greater change in climate since the past), while value close to 0 indicate low climate vulnerability.

We computed the Pearson’s correlation coefficients between climate, anthropogenic and species vulnerabilities to evaluate concordance between these three measures. In order to account for spatial autocorrelation, we computed the significance levels of all the correlation coefficients using modified *t*-test^52^. We performed these analyses in Spatial Analysis in Macroecology (SAM) version 4.0^53^.

### Classification of protected areas

We classified Chinese PA based on three vulnerability indices explained above. We first identified (i) PA with significantly high species vulnerability (species vulnerability hotspots), (ii) PA with significantly high anthropogenic vulnerability (anthropogenic vulnerability hotspots), and (iii) PA with significantly high climate vulnerability (climate vulnerability hotspots). We used a null model approach to assess significance and identify the hotspots of each vulnerability category. We first randomized respective vulnerability scores of each PA. In doing so, we kept a track of where the observed value was greater than the randomized value. We repeated this process 1000 times. The significance of each PA was then assessed by evaluating the rank relative position of the original values against those of the random realizations. For each vulnerability category, we applied a one-tailed test to identify significantly high values. If the observed value was within the highest 5% of the null distribution for that PA, it was identified as significantly high (*p* < 0.05).

We overlaid the spatial maps of these three hotspots in ArcGIS 10.2 and counted the number of overlaps. The PAs with three overlaps, i.e., those with significantly high value for all three vulnerability indices, were classified as “Level 1” PA. The PAs with two overlaps, i.e., those with significantly high value for two out of three vulnerability indices, were classified as “Level 2” PA. The PAs with no overlap, i.e. those with significantly high value for one out of three vulnerability indices, were classified as “Level 3” PA. The protected areas that did not have significantly high values for any of these three vulnerability indices were classified as “Level 4” PA. Level 4 PAs have comparatively low scores for species, climate and anthropogenic vulnerabilities.

### Intensity of climate and human footprint changes in species vulnerability hotspots and coldspots

In order to evaluate the intensity of climate change and human pressure change on the vulnerability hotspots of individual species groups (i.e. birds, mammals, amphibians, reptiles, and plants), we identified the species vulnerability hotspots of each group that, respectively, overlapped with the climate vulnerability hotspots and the anthropogenic vulnerability hotspots. We counted the total number of overlaps to identify (i) the mutual hotspots of species and climate vulnerabilities and (ii) the mutual hotspots of species and anthropogenic vulnerabilities.

We also compared the intensity of climate change and human footprint change (ΔHFP) in the species vulnerability hotspots and coldspots to evaluate if species vulnerability hotspots and coldspots have been under similar influence of climate and human pressure changes since the past. We defined species vulnerability hotspots as the PAs with significantly high species vulnerability and species vulnerability coldspots as the PAs with significantly low species vulnerability. We applied similar randomization approach (see above) to identify hotspots and coldspots of species vulnerability However, we applied a two-tailed test to recover both significantly high and low values. If the observed value was within the highest 2.5% of the null distribution for that PA, it was identified as significantly high (hotspots) and if the observed value was within the lowest 2.5% of the null distribution for that PA, it was identified as significantly low (coldspots).

We compared the differences in climate change and human footprint change between species vulnerability hotspots and coldspots by bootstrapping the samples 1000 times. We calculated the difference in means between hotspots and coldspots in each repetition. Finally, we used this bootstrapped means to create the null distribution and find statistically significant difference in climate change and human footprint change values between species vulnerability hotspots and coldspots.

In order to evaluate the magnitude of future climate warming in each PA, we obtained the future climate data from the worldclim database (http://www.worldclim.org/) at the spatial resolution of 30 arc seconds. We obtained the data simulated from three general circulation models: BCC-CM1-1, CCSM4, and MIROC-ESM and chose the lowest emission scenario (i.e., rcp 2.6) for the year 2070. We calculated the average future mean annual temperature (MAT) from these three data layers using the raster calculator tool in ArcGIS 10.2 and subsequently calculated the average MAT for each PA using the zonal.stats function in the R package ‘spatialEco’^47^. To enable comparison, we also obtained the data for the current climate (MAT) from the worldclim database at the spatial resolution of 30 arc seconds and similarly calculated the average value for each PA. We estimated the change in temperature in each PA by subtracting the future MAT with the current MAT.

### Reporting summary

Further information on research design is available in the Nature Research Reporting Summary linked to this article.

## Supplementary information

Supplementary Information

Description of Additional Supplementary Files

Supplementary Data 1

Reporting Summary

## Data Availability

We obtained the global map of human footprint (HFP) for 1993 and 2009 from https://wcshumanfootprint.org, the monthly minimum temperature, monthly maximum temperature and monthly precipitation of the past (1961–1970) and the present (2010–2019) from the Climate Research Unit (CRU TS v. 4.04) database (http://www.cru.uea.ac.uk/data) and the current and future climate data (mean annual temperature) from the worldclim database (http://www.worldclim.org/). The list of threatened seed plants and vertebrates are provided as Supplementary Data.
