# Author Correction: Vulnerabilities of protected lands in the face of climate and human footprint changes

**DOI:** 10.1038/s41467-021-23423-2

**Published:** 2021-05-10

**Authors:** Nawal Shrestha, Xiaoting Xu, Jiahui Meng, Zhiheng Wang

**Affiliations:** 1 State Key Laboratory of Grassland Agro-Ecosystem, Institute of Innovation Ecology, Lanzhou University, Lanzhou, China; 2 Institute of Ecology and Key Laboratory for Earth Surface Processes of the Ministry of Education, College of Urban and Environmental Sciences, Peking University, Beijing, China; 3 Society for Conservation Biology Nepal, Bagdol, Lalitpur, Nepal; 4 Key Laboratory of Bio-Resource and Eco-Environment of Ministry of Education, College of Life Sciences, Sichuan University, Chengdu, China

**Keywords:** Biodiversity, Conservation biology, Macroecology

Correction to: *Nature Communications* 10.1038/s41467-021-21914-w, published online 12 March 2021.

In the original version of this Article, the maps in Figs. 1, 2, and 4, and Supplementary Figs. 1, 2, 3, and 4 omitted territories in the South China Sea. The maps have now been updated according to the territorial map guidelines of the authors’ institutions in both the PDF and HTML versions of the Article. Springer Nature remains neutral with regard to jurisdictional claims in published maps and institutional affiliations.

## Supplementary information

Supplementary information

