# Supplementary material for: Vulnerabilities of protected lands in the face of climate and human footprint changes
Source: Nat Commun. 2021 Mar 12;12:1632. doi: 10.1038/s41467-021-21914-w (PMC7955075; doi:10.1038/s41467-021-21914-w)
Supplement: Supplementary file 1 — Supplementary Information [file 41467_2021_21914_MOESM1_ESM.pdf]

Supplementary Information

Vulnerabilities of protected lands in the face of climate and human footprint changes

Nawal Shrestha[1,2,3]*, Xiaoting Xu[4], Jiahui Meng[2] & Zhiheng Wang[2]*

[1]State Key Laboratory of Grassland Agro-Ecosystem, Institute of Innovation Ecology, Lanzhou University, Lanzhou, China

[2]Institute of Ecology and Key Laboratory for Earth Surface Processes of the Ministry of Education, College of Urban and Environmental Sciences, Peking University, Beijing, China

[3]Society for Conservation Biology Nepal, Bagdol, Lalitpur, Nepal

[4]Key Laboratory of Bio-Resource and Eco-Environment of Ministry of Education, College of Life Sciences, Sichuan University, Chengdu, China

*Corresponding authors: nawalshrestha@gmail.com (NS); zhiheng.wang@pku.edu.cn (ZW)

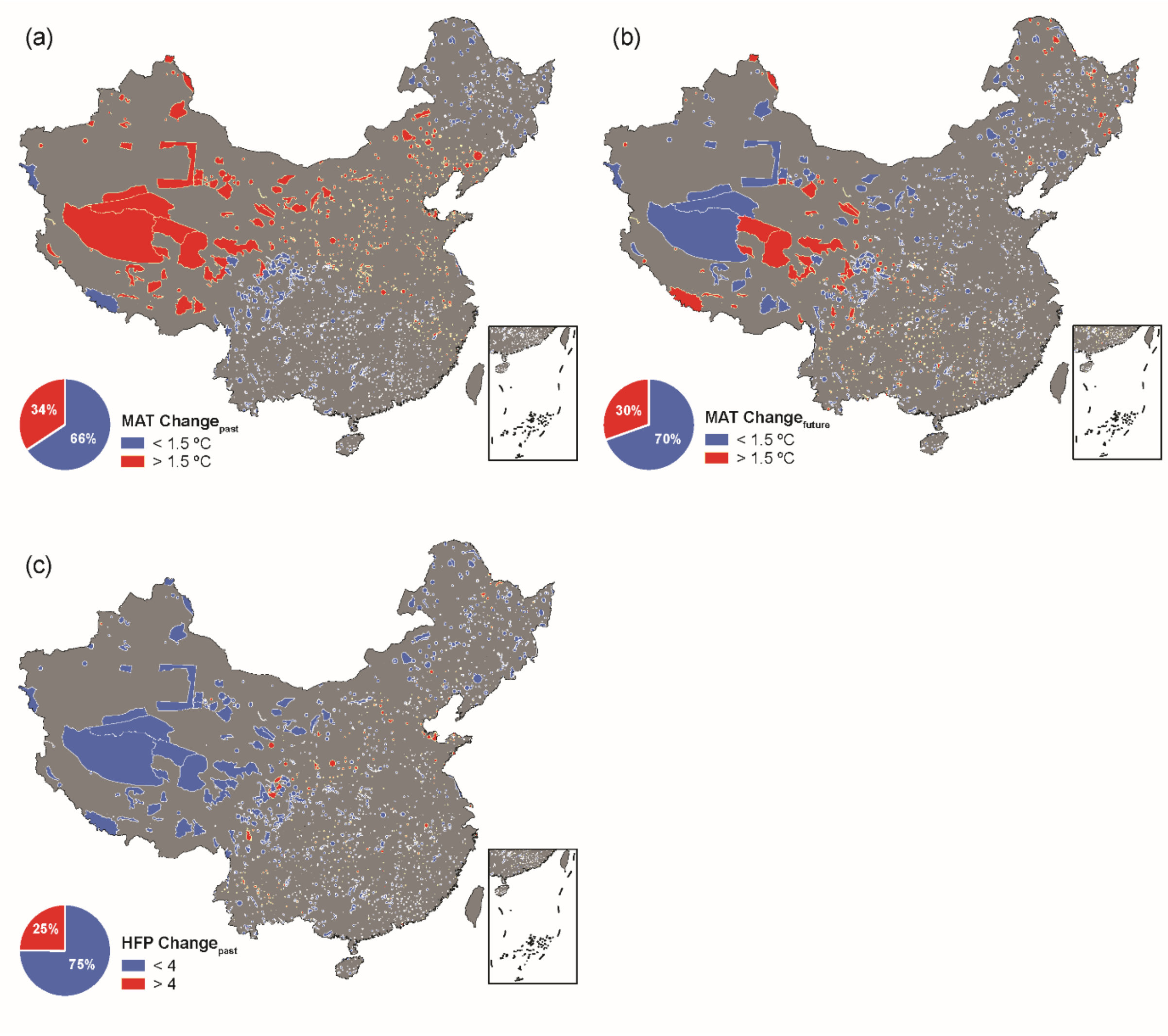

Supplementary Figure 1. Intensity of (a) climate warming in the past, (b) the predicted future warming and (c) change in human footprint in Chinese protected areas. Numbers in the pie chart are the proportions of protected areas in each category.

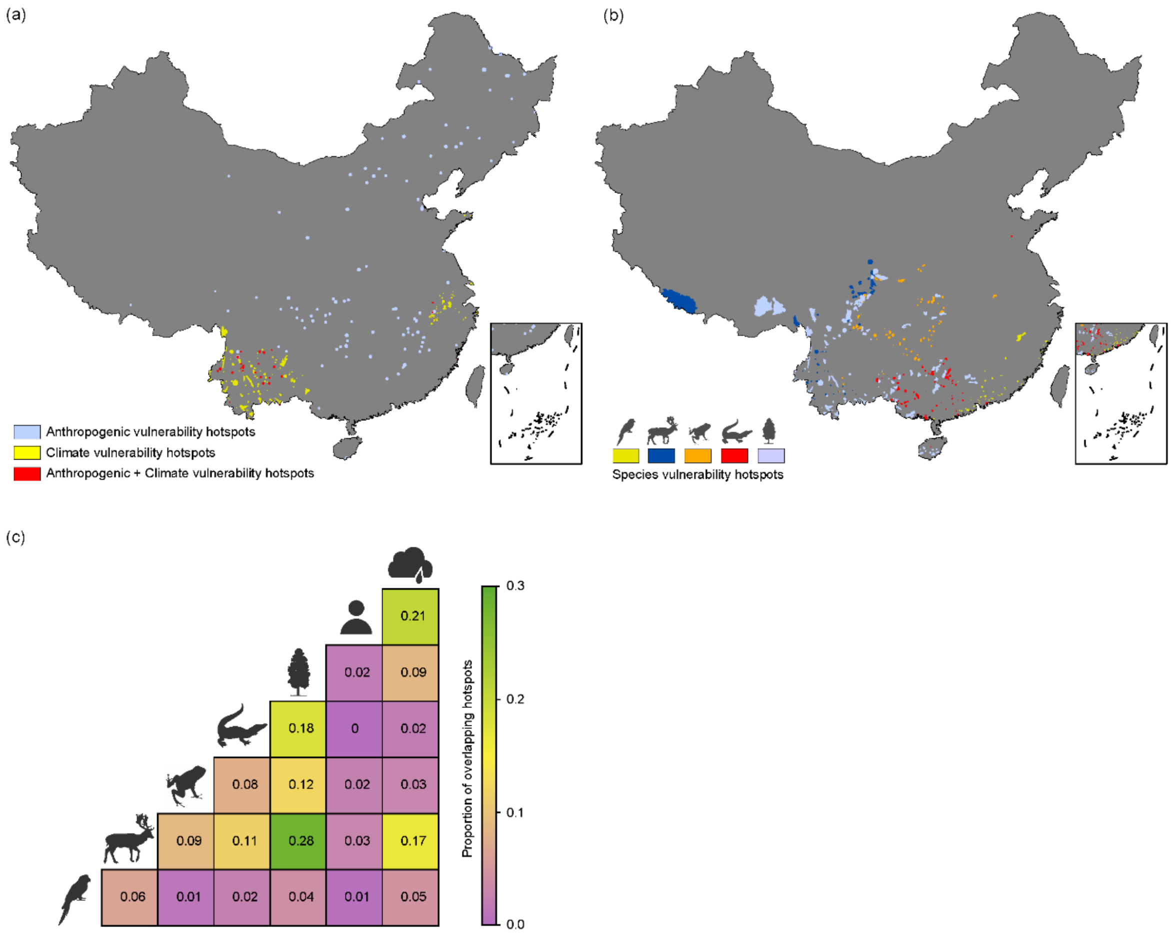

Supplementary Figure 2. Spatial patterns of (a) anthropogenic and climate vulnerability hotspots and (b) species vulnerability hotspots of respective groups. (c) Proportions of overlapping hotspots of each group.

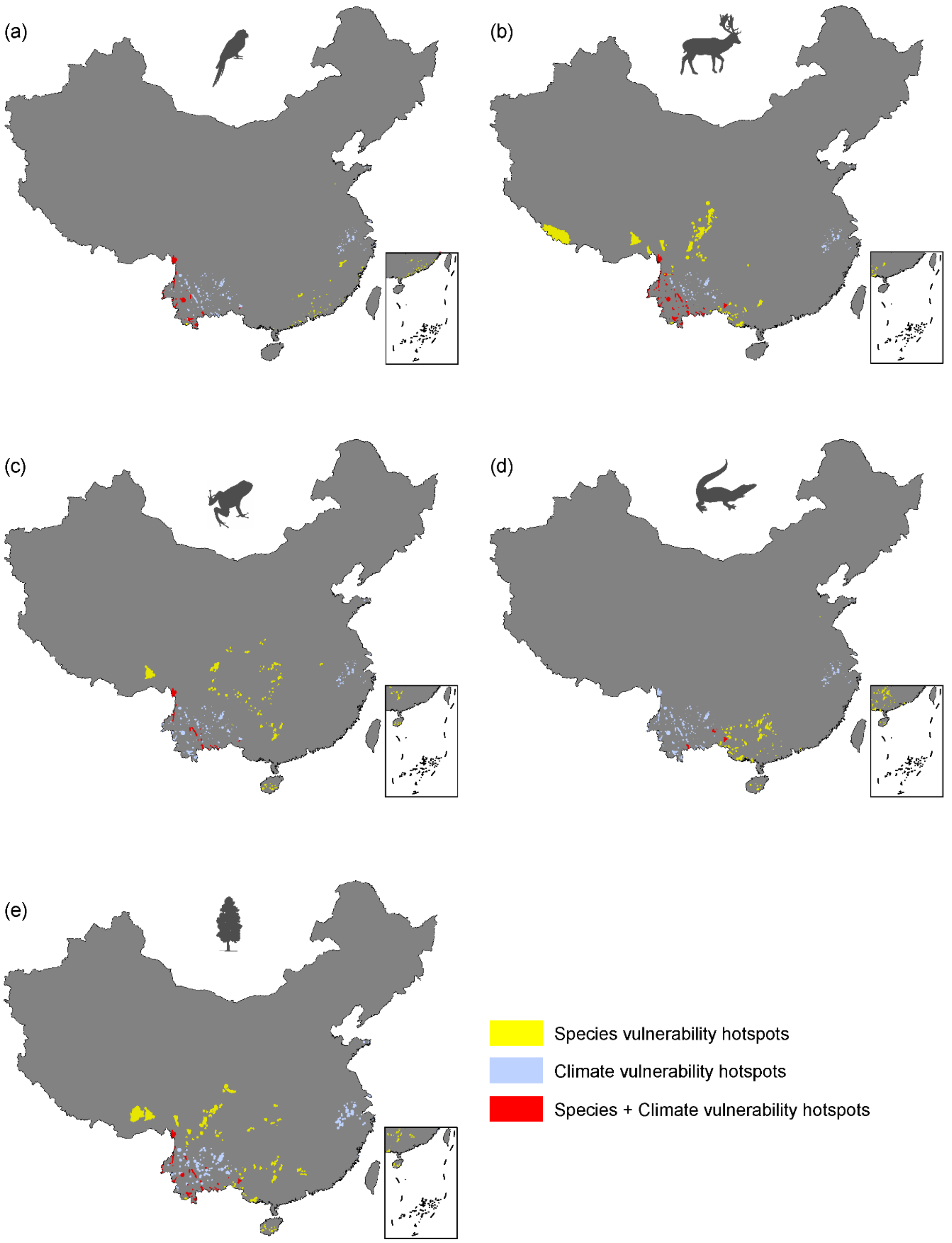

Supplementary Figure 3. Spatial overlap between climate vulnerability hotspots and species vulnerability hotspots of (a) birds, (b) mammals, (c) amphibians, (d) reptiles and (e) plants.

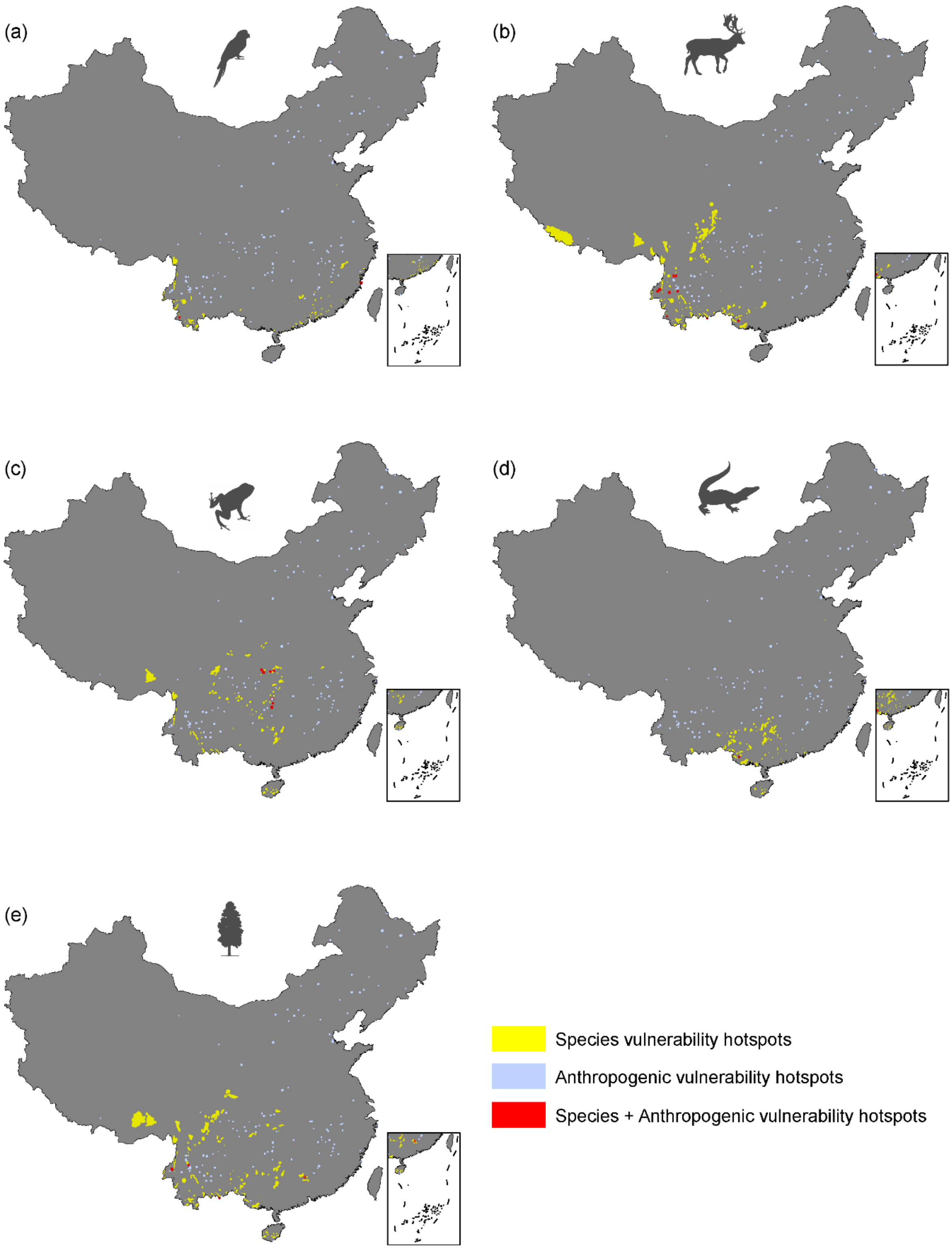

Supplementary Figure 4. Spatial overlap between anthropogenic vulnerability hotspots and species vulnerability hotspots of (a) birds, (b) mammals, (c) amphibians, (d) reptiles and (e) plants.

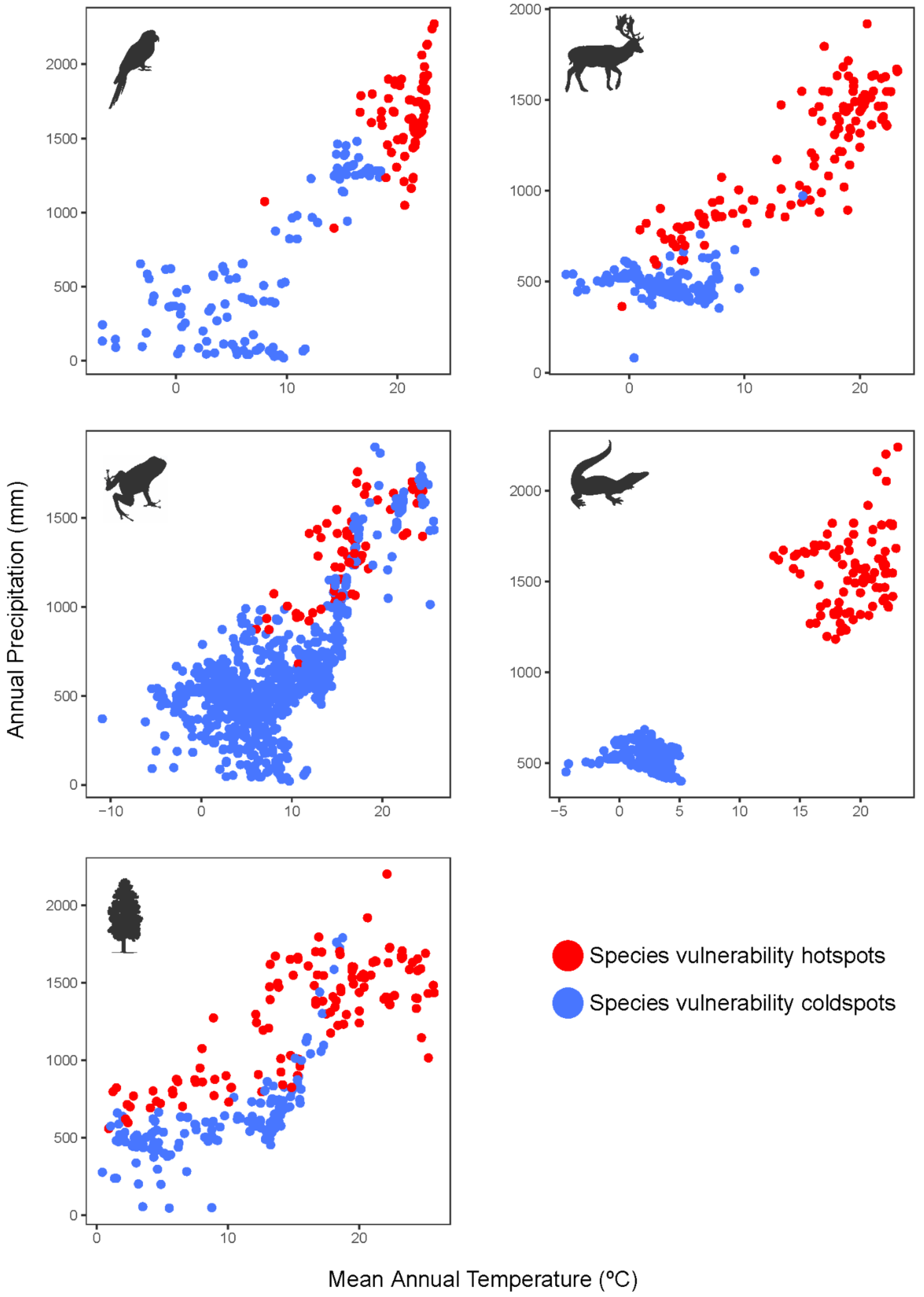

Supplementary Figure 5. Location of species vulnerability hotspots and coldspots of respective groups in a 2-dimensional climate space represented by current mean annual temperature and annual precipitation.

Supplementary Table 1. List of 6 bioclimatic variables (past climate: 1961-1970) used in this study, with loadings on principal components axes 1 and 2 (PCA1 and PCA2). PCA1 and PCA2 explained 82.15% and 7.64% of total variance, respectively.

**Variables**	**PCA1 loadings**	**PCA2 loadings**
Mean annual temperature (MAT)	-0.43416	0.196663
Mean temperature of warmest quarter (MTWQ)	-0.37064	0.806148
Mean temperature of coldest quarter (MTCQ)	-0.41699	-0.10651
Mean annual precipitation (MAP)	-0.43566	-0.30936
Precipitation of wettest quarter (PWQ)	-0.41138	-0.44886
Precipitation of driest quarter (PDQ)	-0.37578	-0.05411

Supplementary Table 2. List of 6 bioclimatic variables (current climate: 2010-2019) used in this study, with loadings on principal components axes 1 and 2 (PCA1 and PCA2). PCA1 and PCA2 explained 81.29% and 8.13% of total variance, respectively.

**Variables**	**PCA1 loadings**	**PCA2 loadings**
Mean annual temperature (MAT)	-0.434	0.334229
Mean temperature of warmest quarter (MTWQ)	-0.36921	0.684023
Mean temperature of coldest quarter (MTCQ)	-0.4139	0.121867
Mean annual precipitation (MAP)	-0.43404	-0.35577
Precipitation of wettest quarter (PWQ)	-0.41808	-0.33798
Precipitation of driest quarter (PDQ)	-0.37528	-0.4059
