# Supplementary material for: Vulnerabilities of protected lands in the face of climate and human footprint changes
Source: Nat Commun. 2021 Mar 12;12:1632. doi: 10.1038/s41467-021-21914-w (PMC7955075; doi:10.1038/s41467-021-21914-w)
Supplement: Supplementary file 2 — Description of Additional Supplementary Files [file 41467_2021_21914_MOESM2_ESM.docx]

**Description of Additional Supplementary Files**

**File Name:** Supplementary Data 1

**Description:** List of threatened taxa evaluated and their conservation status.
