# Supplementary material for: Vulnerabilities of protected lands in the face of climate and human footprint changes
Source: Nat Commun. 2021 Mar 12;12:1632. doi: 10.1038/s41467-021-21914-w (PMC7955075; doi:10.1038/s41467-021-21914-w)
Supplement: Supplementary file 4 — Reporting Summary [file 41467_2021_21914_MOESM4_ESM.pdf]

nature research

Corresponding author(s): Nawal Shrestha

Last updated by author(s): Feb 5, 2021

Reporting Summary

Nature Research wishes to improve the reproducibility of the work that we publish. This form provides structure for consistency and transparency in reporting. For further information on Nature Research policies, see our Editorial Policies and the Editorial Policy Checklist.

Statistics

For all statistical analyses, confirm that the following items are present in the figure legend, table legend, main text, or Methods section.

n/a	Confirmed	
☒	☐	The exact sample size (*n*) for each experimental group/condition, given as a discrete number and unit of measurement
☒	☐	A statement on whether measurements were taken from distinct samples or whether the same sample was measured repeatedly
☐	☒	The statistical test(s) used AND whether they are one- or two-sided<br>*Only common tests should be described solely by name; describe more complex techniques in the Methods section.*
☒	☐	A description of all covariates tested
☒	☐	A description of any assumptions or corrections, such as tests of normality and adjustment for multiple comparisons
☐	☒	A full description of the statistical parameters including central tendency (e.g. means) or other basic estimates (e.g. regression coefficient) AND variation (e.g. standard deviation) or associated estimates of uncertainty (e.g. confidence intervals)
☒	☐	For null hypothesis testing, the test statistic (e.g. *F*, *t*, *r*) with confidence intervals, effect sizes, degrees of freedom and *P* value noted<br>*Give P values as exact values whenever suitable.*
☒	☐	For Bayesian analysis, information on the choice of priors and Markov chain Monte Carlo settings
☒	☐	For hierarchical and complex designs, identification of the appropriate level for tests and full reporting of outcomes
☐	☒	Estimates of effect sizes (e.g. Cohen's *d*, Pearson's *r*), indicating how they were calculated

Our web collection on statistics for biologists contains articles on many of the points above.

Software and code

Policy information about availability of computer code

Data collection	No software was used for data collection. They were directly downloaded from public repositories.
Data analysis	R package 'spatialEco' version 1.2-0, R package 'dismo' version 1.1-4 , ArcGIS version 10.2, R version 4.0.2, Spatial Analysis in Macroecology (SAM) version 4.0.

For manuscripts utilizing custom algorithms or software that are central to the research but not yet described in published literature, software must be made available to editors and reviewers. We strongly encourage code deposition in a community repository (e.g. GitHub). See the Nature Research guidelines for submitting code & software for further information.

Data

Policy information about availability of data

All manuscripts must include a data availability statement. This statement should provide the following information, where applicable:

- Accession codes, unique identifiers, or web links for publicly available datasets
- A list of figures that have associated raw data
- A description of any restrictions on data availability

We obtained the global map of human footprint (HFP) for 1993 and 2009 from https://wcshumanfootprint.org, the monthly minimum temperature, monthly maximum temperature and monthly precipitation of the past (1961-1970) and the present (2010-2019) from the Climate Research Unit (CRU TS v. 4.04) database (http://www.cru.uea.ac.uk/data) and the current and future climate data (mean annual temperature) from the worldclim database (http://www.worldclim.org/). The list of threatened seed plants and vertebrates are provided as Supplementary Data.

Field-specific reporting

Please select the one below that is the best fit for your research. If you are not sure, read the appropriate sections before making your selection.

☐ Life sciences ☐ Behavioural & social sciences ☒ Ecological, evolutionary & environmental sciences

For a reference copy of the document with all sections, see nature.com/documents/nr-reporting-summary-flat.pdf

Ecological, evolutionary & environmental sciences study design

All studies must disclose on these points even when the disclosure is negative.

Study description	Assess the vulnerabilities of protected areas using multiple dimensions, including biodiversity, climate change and human pressure.
Research sample	The research sample includes 103 birds, 86 mammals, 134 amphibians, 50 reptiles and 2,983 plants in China. The distributions of threatened vertebrates (birds, mammals, amphibians and reptiles) were obtained from the IUCN's Red List (http://www.iucnredlist.org), while that of plants were obtained from Flora of China, Atlas of woody plants in China, provincial and local floras, checklists of nature reserves, various inventory reports across China and peer-reviewed papers. The climate data (monthly minimum temperature, monthly maximum temperature and monthly precipitation) of the past (1961-1970) and the present (2010-2019) were obtained from Climate Research Unit (CRU TS v. 4.04) database (http://www.cru.uea.ac.uk/data) at the spatial resolution of 0.5° x 0.5°, current and future temperature data (mean annual temperature) were obtained from the worldclim database (http://www.worldclim.org) at the spatial resolution of 30 arc seconds, and the human footprint data was obtained from https://wcshumanfootprint.org at the spatial resolution of 1 km x 1 km.
Sampling strategy	Range maps of all the available species, climate data and human footprint data of specified time periods were used.
Data collection	Data were directly downloaded from public repositories. The distributions of threatened vertebrates (birds, mammals, amphibians and reptiles) were downloaded from www.iucnredlist.org (July 20, 2020) as GIS shapefiles. The distribution data of plants was compiled from Flora of China, Atlas of woody plants in China, provincial and local floras, checklists of nature reserves, various inventory reports across China and peer-reviewed papers. The global rasters of climate (monthly minimum temperature, monthly maximum temperature and monthly precipitation) of the past (1961-1970) and the present (2010-2019) were downloaded from http://www.cru.uea.ac.uk/data (October 6, 2020). The global rasters of current and future temperature (mean annual temperature) were downloaded from http://www.worldclim.org/ (December 20, 2020). The global rasters of human footprint (1993 and 2009) were downloaded from https://wcshumanfootprint.org (November 13, 2019). The average value of all the rasters (climate, temperature and human footprint) was calculated for each protected area in China using zonal.stats function in the R package 'spatialEco'. The number of species in each protected area was estimated by intersecting the range maps of species with the polygons of protected areas in ArcGIS 10.2.
Timing and spatial scale	Climate data of two time periods: 1961-1970, 2010-2019 and temperature data (mean annual temperature) of two time periods: current and 2070 (rcp 2.6) at the global scale, human footprint data of 1993 and 2009 at the global scale and contemporary distribution data of threatened vertebrates and plants in China.
Data exclusions	None excluded.
Reproducibility	In order to check the accuracy of our findings, we reanalyzed everything 3 times and all the attempts produced identical results. Because our study was based on the datasets obtained from secondary sources and involved static variables, the analysis output is not likely to change no matter how many repetitions are performed.
Randomization	In order to identify the hotspot protected areas (PA) of respective vulnerabilities, the vulnerability score of each PA was randomized 1000 times. The significance of each PA was then assessed by evaluating the rank relative position of the original values against those of the random realizations. We also compared the differences in climate change and human footprint change between species vulnerability hotspots and coldspots by bootstrapping the climate and human footprint data 1000 times.
Blinding	The data we collected and the analyses we performed did not include any confirmation bias. We neither had prior assumptions on the structure of the data nor the output of the analyses. Our study was not conducted to validate or test a priori hypotheses. The output was completely unknown to us before the study was undertaken. Therefore, blinding was not directly relevant to our study. We collected all the available data needed for our study from publicly available databases and performed the analyses and presented the findings without altering anything.

Did the study involve field work? ☐ Yes ☒ No

Reporting for specific materials, systems and methods

We require information from authors about some types of materials, experimental systems and methods used in many studies. Here, indicate whether each material, system or method listed is relevant to your study. If you are not sure if a list item applies to your research, read the appropriate section before selecting a response.

Materials & experimental systems

n/a	Involved in the study
☒	☐ Antibodies
☒	☐ Eukaryotic cell lines
☒	☐ Palaeontology and archaeology
☒	☐ Animals and other organisms
☒	☐ Human research participants
☒	☐ Clinical data
☒	☐ Dual use research of concern

Methods

n/a	Involved in the study
☒	☐ ChIP-seq
☒	☐ Flow cytometry
☒	☐ MRI-based neuroimaging
